# The Association between Changes in External Environment Caused by Migration and Inappropriate Antibiotic Use Behaviors among Chinese University Students: A Cross-Sectional Study

**DOI:** 10.3390/antibiotics8040200

**Published:** 2019-10-28

**Authors:** Jingjing Lu, Xiaomin Wang, Leesa Lin, Ziming Xuan, Yanhong Jessika Hu, Xudong Zhou

**Affiliations:** 1 Institute of Social Medicine, School of Medicine, Zhejiang University, Hangzhou 310058, China; jingjinglu@zju.edu.cn (J.L.); xiaominwang2018@zju.edu.cn (X.W.); 2 Department of Public Health, Environments and Society, Faculty of Public Health and Policy, London School of Hygiene & Tropical Medicine, London WC1H 9SH, UK; leesaklin@gmail.com; 3 Department of Global Health and Social Medicine, Harvard Medical School, Boston, MA 02115, USA; 4 Department of Community Health Sciences, Boston University School of Public Health, Boston, MA 02118, USA; zxuan@bu.edu; 5 Murdoch Children’s Research Institute, University of Melbourne. Royal Children’s Hospital, Melbourne VIC 3052, Australia; huhubest@gmail.com

**Keywords:** antibiotic use, external factor, university student

## Abstract

Objectives: This study aims to explore how changes in external factors caused by migration impact antibiotic use behaviors among Chinese university students in comparison to their peers from host areas and origin areas. Migration status was determined by host universities and origin areas, which were broadly defined as eastern vs. western regions in China. Methods: This study analyzed secondary data from a cross-sectional study conducted in China about the antibiotic use behaviors of university students in 2015. Students were divided into four groups: eastern local students (E-Es), western local students (W-Ws), eastern–western migrant students (E-Ws), and western–eastern migrant students (W-Es). Results: After controlling for gender, grade, major, hometown (rural or urban), and parents’ education, E-Ws reported a significantly higher odds of asking for antibiotics (OR = 2.13; 95% CI = 1.54–3.03; *p* < 0.001) and taking antibiotics prophylactically (OR = 1.85; 95% CI = 1.32–2.56; *p* < 0.001) compared with E-Es; W-Es reported a significantly lower odds of asking for antibiotics (OR = 0.56; 95% CI = 0.37–0.83; *p* < 0.01) and taking antibiotics prophylactically (OR = 0.57; 95% CI = 0.41–0.81; *p* < 0.01) compared with W-Ws. Discussion: Regional differences likely interacted with students’ migration status in forming different antibiotic use behaviors. Factors including financial incentives and loose regulations of antibiotic over-prescription by health providers and peer influence may contribute to worsened antibiotic use behaviors among E-Ws.

## 1. Introduction

Antibiotics are among the most commonly used drugs worldwide [1]. However, the effect of antibiotics against bacterial infections has been greatly undermined due to the increase in antimicrobial resistance (AMR). AMR can cause high mortality rates of common diseases, such as pneumonia [2], and increase the financial burden of health expenditure. Bacteria bearing resistance to one or more antimicrobials from at least three different antimicrobial classes, defined as multidrug resistance (MDR) bacteria, has spread surprisingly fast globally, especially in healthcare institutions [3], which can lead to prolonged hospitalization and associated costs [4]. Notably, the acronym “ESKAPE pathogens” refers to the most frequently reported MDR, including *Enterococcus faecium*, *Staphylococcus aureus*, *Klebsiella pneumoniae*, *Acinetobacter baumanii*, *Pseudomonas aeruginosa*, and *Enterobacter species*, emphasizing that they are responsible for the majority of hospital infections and are impervious to the effects of antibiotics [5]. One international review on AMR in 2014 estimated that annual mortality attributable to AMR was 700,000, and this number may rise to 10 million by 2050 if action is not taken to reduce inappropriate use of antibiotics [6]. Inappropriate antibiotic use behaviors, such as self-medication with antibiotics (SMA), which can increase the selection of resistant bacteria [7], are recognized as the most important factor that is responsible for increasing AMR [1,8].

With a great interest in inappropriate antibiotic use behaviors and the subsequent increase in AMR, many international studies have evaluated the factors related to inappropriate antibiotic use behaviors. Factors that may influence antibiotic use behaviors can be divided into internal and external factors [9]. For the purpose of this study, internal factors are personal factors, including knowledge, awareness, and attitudes that can influence one’s behaviors. External factors are environmental determinants, including social norms, policies, and regulations that impact the supply side, such as health care providers and pharmacies [10]. The effects of internal factors on inappropriate antibiotic use behaviors, including knowledge, attitudes, and beliefs regarding proper antibiotic use [11,12], have been well documented worldwide, including poor knowledge, irrational attitudes, and beliefs. Apart from the internal factors mentioned above, external factors, including social norms and regulatory environments, also deserve attention. However, reports on the effects of poor external factors, including loose regulations on inappropriate antibiotic use, have been limited in scope [13].

The antibiotic use behaviors of migrants have drawn great attention because this population experiences different social norms and environmental determinants compared to sedentary populations. Thus, the migrant population might report unique antibiotic use patterns due to changes in external factors. One study conducted in the United States found that compared with the local population, migrants were 17% more likely to expect antibiotics from a doctor [14]. It has also been reported that Latino immigrants in the United States may use non-prescribed antibiotics more frequently due to financial and sociocultural barriers [15,16]. Compared with local Australians, Chinese migrants in Australia were more likely to carry SMA out of dissatisfaction with general practitioner services [17]. And immigrants in the Netherlands received more antibiotics from clinics than non-immigrants did [18]. However, all of these studies mentioned above were only able to describe the difference of inappropriate antibiotic use behaviors between the immigrant population and the local population of host countries. Yet, no study has described the difference of antibiotic use behaviors between the migrant population and the local population of origin countries.

Inappropriate antibiotic use is highly pervasive [19,20] and varies greatly in China, where eastern provinces report a lower prevalence of inappropriate antibiotic use than other provinces [21]. Generally speaking, eastern China is far more developed than western China, resulting in more conducive factors related to appropriate antibiotic use behaviors. Regarding internal factors, those living in eastern China reported significantly better health literacy [22] and antibiotic knowledge and attitudes than those in western China [23]. Regarding external factors, policies that constrain antibiotic overuse in hospitals and pharmacies [24] performed better in eastern China. Analysis of one antibiotic stewardship program in hospitals showed that this program had greater effects in eastern China than western China [25]. Similarly, reports demonstrated that pharmacies in eastern China were under stricter regulations on over-the-counter sales of antibiotics [26].

For the present study, we used secondary data from a cross-sectional study conducted in six provinces of China about antibiotic use behaviors of university students conducted from September to November 2015 [27]. This study aimed to explore the knowledge and healthcare-seeking behaviors of university students from across China and determine the association between this knowledge and healthcare-seeking behaviors in relation to antibiotic use. From this large scale cross-sectional study, researchers found that massive misuse of antibiotics for self-limiting illnesses by well-educated young adults was a serious concern.

The present study aimed to explore the association between migration across geographic regions and inappropriate antibiotic use behaviors among Chinese university students with a comparison with peers from origin areas. Based on the aforementioned literature, two major research questions were assessed: 1) how would antibiotic behaviors change among eastern students who were from areas with excellent external factors and migrated to western areas that with poor external factors; 2) how would antibiotic behaviors change among western students who were from areas with poor external factors and migrated to eastern areas that with excellent external factors.

## 2. Results

Among the six sampled provinces, Zhejiang and Tianjin were in the eastern developed region of China while Guizhou and Gansu were in the western developing region. Zhejiang University was selected from Zhejiang Province, Nankai University from Tianjin, Guizhou University from Guizhou, and Lanzhou University from Gansu. The students in the four universities who originated from the above four provinces were included in this study. (Table 1)

**Figure 1:**
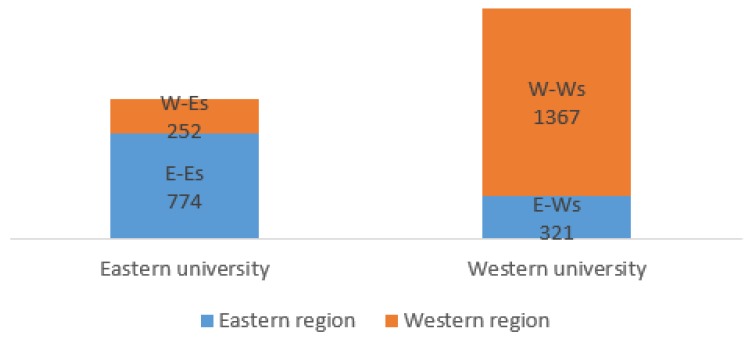
The grouping of university students for the present study. Note: E-Es: Eastern local students; W-Ws: Western local students; E-Ws: Eastern–western migrant students; W-Es: Western–eastern migrant students.

As shown in Table 1 and Figure 1, the students were divided into four groups: E-Es were students from eastern regions studying at eastern universities; W-Ws were students from western regions and studying at western universities; E-Ws were students from eastern regions but studying at western universities; W-Es were students from western regions but studying at eastern universities. As Table 1 shows, a total number of 2714 university students were included in this study with 774 E-Es, 252 W-Es, 321 E-Ws, and 1367 W-Ws.

Males (49.15%) and females (50.85%) were equally represented, so were students in their sophomore (32.72%), junior (32.57%), and senior (34.71%). Students majoring in social science (55.82%) outnumbered science (44.18%). Roughly half (49.74%) of the students were from rural areas. One-third of students (32.79%) reported their parents’ education level to be college or above, 22.99% reported to be high school, and 44.22% reported to be middle school or below. About one-fifth of (19.34%) students reported asking their doctors for antibiotics in the past year. A quarter of (25.83%) students reported taking antibiotics prophylactically (e.g., for the common cold) in the past year. Table 2 shows significant differences by group status with respect to gender, grade, parents’ education level, and inappropriate antibiotic use behaviors (asking for antibiotics and taking antibiotics prophylactically) (*p* < 0.01). W-Ws reported the highest prevalence of asking for antibiotics (31.53%), followed by E-Ws (26.79%), W-Es (21.43%), and E-Es (16.80%). E-Ws reported the highest prevalence of taking antibiotics prophylactically (25.55%), followed by W-Ws (21.58%), E-Es (14.49%), and W-Es (14.29%).

**Figure 2:**
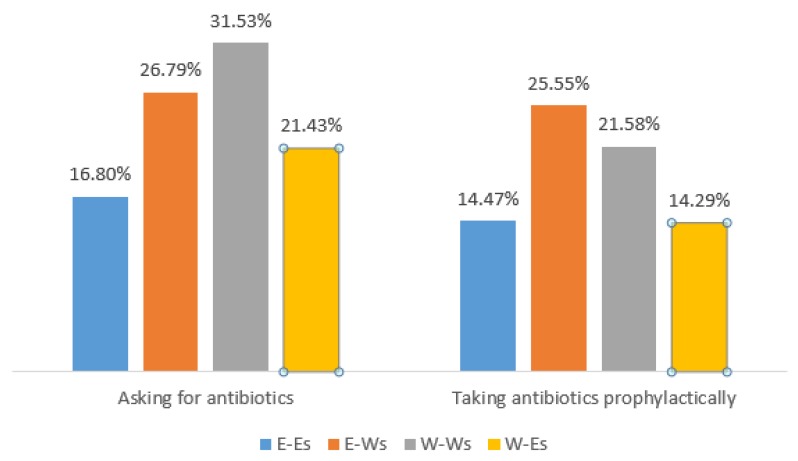
Descriptive statistics of antibiotic use behaviors among university students by groups.

Figure 2 and Table 3 show the correlations between migration and antibiotic use behaviors among university students. Significant and positive correlations were found between E-W migration and asking for antibiotics (*r* = 0.07, *p* < 0.01) as well as taking antibiotics prophylactically (*r* = 0.08, *p* < 0.01). Significant and negative correlations were found between W-E migration and asking for antibiotics (*r* = −0.13, *p* < 0.01) as well as taking antibiotics prophylactically (*r* = −0.11, *p* < 0.01).

Table 4 shows the results of the logistic regression analyses of changes in antibiotic use behaviors among students who were from eastern regions or attended eastern universities. After controlling for gender, grade, major, hometown, parents’ education, E-Ws reported a significantly higher odds of asking for antibiotics (OR = 2.13; 95% CI = 1.54–3.03; *p* < 0.001) and taking antibiotics prophylactically (OR = 1.85; 95% CI = 1.32–2.56; *p* < 0.001) while W-Es reported no significant difference in the odds of asking for antibiotics or taking antibiotics prophylactically compared with E-Es.

Table 5 shows the results of the logistic regression analyses of changes in antibiotic use behaviors among students who were from western regions or attended western universities. After controlling for gender, grade, major, hometown, and parents’ education level, W-Es reported a significantly lower odds of asking for antibiotics (OR = 0.56; 95% CI = 0.37–0.83; *p* < 0.01) and taking antibiotics prophylactically (OR = 0.57; 95% CI = 0.41–0.81; *p* < 0.01) while E-Ws reported no significant difference in the odds of asking for antibiotics or taking antibiotics prophylactically compared with W-Ws.

## 3. Discussion

To our knowledge, this is the first study to assess the association between migration across geographic regions and antibiotic use behaviors among Chinese university students. Through the comparison of antibiotic use behaviors among E-Es, W-Ws, E-Ws, and W-Es, we found that antibiotic use behaviors of university students were affected by changes in external environments caused by migration. Interestingly, E-Ws reported poorer antibiotic use behaviors compared with eastern local students, and W-Es reported better antibiotic use behaviors compared with western local students.

University students’ antibiotic use behaviors varied greatly with regard to their regions (See Table 2). The prevalence of asking for antibiotics from doctors among western local university students (31.53%) was almost as double as that of eastern local university students (16.80%). Similar differences can also be observed in the prevalence of taking antibiotics prophylactically without doctor’s advice (21.58% in western local university students vs. 14.47% in eastern local university students). Our results were consistent with the study conducted in southern China, which reported that university students from different provinces reported different SMA rates [28]. However, that study only enrolled local students as a reference group and failed to present the impacts of migration on other non-local students in detail.

Our study revealed that migrant university students, including E-Ws and W-Es, not only reported significantly different antibiotic use behaviors than did their peers in their local areas, but also reported behavioral patterns that conformed to the host area (See Table 4 and Table 5). Migration can be a profound phenomenon that is capable of reshaping societal structures in numerous countries and regions, and one major factor leading to this pattern is acculturation [29]. Acculturation refers to changes in attitudes and behavioral patterns that occur when individuals who migrate to a new area come into continuous contact with the host population and society [30]. The process of acculturation takes place over time, and it involves cultural groups with different characteristics that may affect an individual’s health and health behavior choices [31]. Relevant studies have provided robust evidence for the existence of a correlation between acculturation and subsequently-changed health behaviors, including prescription drug abuse [32], drinking [33], and smoking [34] among immigrants living in the United States. Thus, acculturation may play an important role in the changes in antibiotic use behaviors among these migrant university students, which warrants further investigation.

In addition to acculturation, factors related to the supply side of the health system warrant further discussion. As previously noted, health-care providers in China rely heavily on drug sales to be profitable, which inevitably accounts for antibiotic over-prescription in hospitals and community health facilities [35]. Compared with more developed provinces, healthcare providers in less developed provinces experience more loosened regulations and are more likely to profit from antibiotic over-prescription [36]. One systematic review noted out that the antibiotic over-prescription was more prevalent in the less developed area of China [32], suggesting that health providers in less-developed areas were more likely to prescribe patients with unnecessary antibiotics due to misaligned economic incentives [37]. In the present study, university students’ habits of asking for antibiotics during medical consultations may result in health-providers’ endorsement.

Over-prescription of antibiotics may also be associated with the training and experiences of doctors. It must be mentioned that doctors in the eastern areas, on average, may have more training than those in the western areas [32]. Moreover, hospitals in western China also tend to be associated with higher antibiotic use than hospitals in eastern China [32]. Doctors in less-developed areas in China are also more likely to prescribe antibiotics due to patients’ desires, even though they may be initially unwilling to do so [38]. The improved training could help clinicians recognize the importance of rational antibiotic use [39]. Attention should be paid to students’ demands for antibiotics at health facilities in less-developed areas, which may lead to greater antibiotic over-prescription by doctors.

Easy acquisition of antibiotics from pharmacies without a prescription [40] coupled with the common misconception in China that antibiotics are panaceas [41] contribute to SMA behaviors, including using antibiotics as prophylaxis. In the present study, E-Ws were far more likely (OR = 1.85; 95% CI = 1.35, 2.56) to take antibiotics prophylactically (such as a common cold) compared with their peers who stayed in eastern regions (E-Es). Meanwhile, the use of antibiotics as prophylaxis among W-Es was significantly lower (OR = 0.57; 95% CI = 0.41, 0.81) when compared to W-Ws. As stated above, the effects of relevant antibiotic regulations depend greatly on local governance capabilities, with pharmacies in western China reporting poorer supervision [22]. This suggests that regional disparities in regulations on the sales of antibiotics in pharmacies in China might have affected university students’ antibiotic behaviors significantly in the present study.

Peer influence also may play an important role in the case of inappropriate antibiotic use behaviors in university students. University students are among the highest risk subgroups of emerging adults in terms of prescription drug misuse [42]. University students who were living in the same dorm or who were close friends would share their medicine and experiences with each other [43,44], which may partially account for the fact that E-Ws and W-Es reported behavioral patterns that cohered with the host area.

Our findings highlight the importance of developing a supportive and supervised environment for rational antibiotic use, including the enforcement of relevant regulations on hospitals and pharmacies and advocating for greater adoption of appropriate social norms through public health education. As university students in China complete most of their health-seeking behaviors at hospitals and pharmacies inside their campuses, university health staff should be well trained in rational antibiotic use first and adhere to medication guidelines to limit students’ access to antibiotics.

These findings should be viewed in the context of several limitations: First, this study was conducted from September to November 2015 to measure students’ antibiotic use behaviors in the past year. The prevalence of asking for antibiotics and taking antibiotics prophylactically may, therefore, be underestimated due to recall bias. Second, students’ SMA behaviors were not directly measured, which may limit the generalization of our findings. Third, this study failed to measure external factors directly. Our deduction was based on previous descriptive studies. Finally, this was a cross-sectional study, and therefore, whether the effects of changes in external factors on antibiotic use behavior would last until after these students graduate and migrate to other areas or return to their hometown remain unknown.

## 4. Materials and Methods

### 4.1. Data Collection

A cluster sampling method was adopted. At each university, students attending class on the main campus on the day of the survey were included. At each university, three investigators approached teachers, explained the aim of our survey, and asked for permission to speak to students before the class began. The investigator then explained the aim of the survey to the students, disseminated the printed QR code of the electronic questionnaires, and explained to students how to complete the electronic questionnaire. The study was reviewed and approved by the School of Public Health at Zhejiang University.

### 4.2. Measures

The inappropriate antibiotic use behaviors were assessed with the following questions: “Did you ask your doctors for antibiotics when doctors didn’t intend to prescribe in the past year?” and “Did you take antibiotics to prevent disease (such as a common cold) in the past year?” 

By the period the survey was conducted, freshmen were only enrolled at universities for less than 3 months. Thus, freshmen were not included in the present study since their antibiotic use behaviors in the past year may not happen in the sample provinces.

### 4.3. Statistical Analysis

Data were analyzed using the SPSS version 20.0 and assumed a statistical significance level of *p* < 0.05. We conducted Chi-square tests to examine students’ demographic and inappropriate antibiotic use behaviors. Logistic regressions models were applied to examine the associations between students’ group status and inappropriate antibiotic use behaviors adjusting for gender, grade, major, hometown, and parents’ education. Students’ inappropriate antibiotic behaviors were included as dependent variables (0 = no, 1 = yes), while students’ groups were examined as independent variables.

## 5. Conclusions

External factors played a vastly more important role in forming appropriate antibiotic use behaviors when compared with internal factors in the present study. Factors including financial incentives and loose regulations on antibiotic over-prescription by health providers, and peer influence all attributed to worsened antibiotic use behaviors among E-Ws. To address this problem, western universities should pay greater attention to constructing a supportive and supervised environment for rational antibiotic use and limiting students’ access to antibiotics by educating health staff working at university hospitals and pharmacies.

## Figures and Tables

**Table 1:** The distribution of undergraduate students (*n* = 2714).

Areas	Zhejiang and Nankai University (Eastern Universities)	Guizhou and Lanzhou University (Western Universities)
Zhejiang and Tianjin Province (Eastern Areas)	774 (E-Es)	321 (E-Ws)
Guizhou and Gansu Province (Western Areas)	252 (W-Es)	1367 (W-Ws)

**Table 2:** Descriptive statistics for students stratified by regions and universities.

Variables	E-Es *N* = 774 (%)	W-Es *N* = 252 (%)	E-Ws *N* = 321 (%)	W-Ws *N* = 1367 (%)	χ^2^	*p*
Gender					15.31	0.002
Male	357 (46.12)	130 (51.59)	188 (58.57)	659 (48.21)		
Female	417 (53.88)	122 (48.41)	133 (41.43)	708 (51.79)		
Grade					147.9	<0.001
Sophomore	341 (44.06)	119 (47.22)	105 (32.71)	323 (23.63)		
Junior	248 (28.05)	80 (31.75)	95 (29.60)	461 (33.72)		
Senior	185 (23.90)	53 (21.03)	121 (37.69)	583 (42.65)		
Major					6.226	0.101
Social Science	460 (59.43)	132 (52.38)	177 (55.14)	746 (54.57)		
Science	314 (40.57)	120 (47.62)	144 (44.86)	621 (45.43)		
Hometown					393.5	<0.001
Rural	199 (25.71)	78 (30.95)	147 (45.79)	926 (67.74)		
Urban	575 (74.29)	174 (69.05)	174 (54.21)	441 (32.26)		
Parents’ Education Level					484.8	0.000
Middle School or Below	176 (22.74)	62 (24.60)	103 (32.09)	859 (62.84)		
High School	190 (24.55)	49 (19.44)	99 (30.84)	286 (20.92)		
College or Above	408 (52.71)	141 (55.95)	119 (37.07)	222 (16.24)		
Asking for Antibiotics					28.21	0.000
Yes	130 (16.80)	54 (21.43)	86 (26.79)	431 (31.53)		
No	644 (83.20)	198 (78.57)	235 (73.21)	936 (68.47)		
Taking Antibiotics Prophylactically					58.85	0.000
Yes	112 (14.47)	36 (14.29)	82 (25.55)	295 (21.58)		
No	662 (85.53)	216 (85.71)	239 (74.45)	1072 (78.42)		

**Table 3:** Correlation coefficients between migration and antibiotic use behaviors among university students.

Variables	Asking for Antibiotics	Taking Antibiotics Prophylactically
E-W Migration	0.07 **	0.08 **
W-E Migration	−0.13 **	−0.11 **

* *p* < 0.05, ** *p* < 0.01, *** *p* < 0.001.

**Table 4:** Changes in antibiotic use behaviors among students who were from the eastern region or attended eastern universities.

Variables	Asking for Antibiotics OR (95% CI)	Taking Antibiotics Prophylactically OR (95% CI)
Group		
E-Es	1.00	1.00
W-Es	1.01 (0.67–1.52)	1.39 (0.97–2.00)
E-Ws	2.13 (1.54–3.03) ***	1.85 (1.35–2.56) ***
Gender		
Male	1.00	1.00
Female	0.82 (0.60–1.10)	0.98 (0.74–1.30)
Grade		
Sophomore	1.00	1.00
Junior	0.85 (0.61–1.19)	0.95 (0.69–1.30)
Senior	1.19 (0.82–1.74)	1.11 (0.78–1.57)
Major		
Social Science	1.00	1.00
Science	1.49 (1.09–2.04) *	1.55 (1.16–2.08) **
Hometown		
Rural	1.00	1.00
Urban	1.23 (0.82–1.85)	1.20 (0.82–1.76)
Parents’ Education Level		
Middle School or Below	1.00	1.00
High School	0.86 (0.56–1.33)	0.83 (0.55–1.24)
College or Above	0.80 (0.50–1.27)	0.80 (0.52–1.24)

* *p* < 0.05, ** *p* < 0.01, *** *p* < 0.001.

**Table 5:** Changes in antibiotic use behaviors among students who were from the western region or attended western universities.

Variables	Asking for Antibiotics OR (95% CI)	Taking Antibiotics Prophylactically OR (95% CI)
Group		
W-Ws	1.00	1.00
E-Ws	1.18 (0.88–1.59)	0.78 (0.58–1.03)
W-Es	0.56 (0.37–0.83) **	0.57 (0.41–0.81) **
Gender		
Male	1.00	1.00
Female	0.87 (0.69–1.10)	0.87 (0.70–1.07)
Grade		
Sophomore	1.00	1.00
Junior	0.87 (0.65–1.16)	1.05 (0.82–1.36)
Senior	0.94 (0.71–1.25)	1.18 (0.92–1.51)
Major		
Social Science	1.00	1.00
Science	1.19 (0.94–1.50)	1.44 (1.16–1.78) **
Hometown		
Rural	1.00	1.00
Urban	1.08 (0.81–1.45)	1.20 (0.92–1.56)
Parents’ Education Level		
Middle School or Below	1.00	1.00
High School	0.77 (0.57–1.02)	0.79 (0.61–1.02)
College or Above	0.70 (0.49–0.99) *	0.82 (0.59–1.13)

* *p* < 0.05, ** *p* < 0.01, *** *p* < 0.001.
